# Improving Power Quality in Grid-Connected Photovoltaic Systems: A Comparative Analysis of Model Predictive Control in Three-Level and Two-Level Inverters

**DOI:** 10.3390/s23187901

**Published:** 2023-09-15

**Authors:** Saliha Gada, Arezki Fekik, Miroslav Mahdal, Sundarapandian Vaidyanathan, Ahmed Maidi, Ali Bouhedda

**Affiliations:** 1 Laboratoire de Conception et Conduite des Systèmes de Production, Faculté de Génie Électrique et d’Informatique, Université Mouloud Mammeri, Tizi-Ouzou 15000, Algeria; saliha.gada@fgei.ummto.dz (S.G.); ahmed.maidi@ummto.dz (A.M.); 2 Department of Electrical Engineering, University Akli Mohand Oulhadj-Bouria, Rue Drissi Yahia Bouira, Bouira 10000, Algeria; a.fekik@univ-bouira.dz (A.F.); a.bouhedda@univ-bouira.dz (A.B.); 3 Department of Control Systems and Instrumentation, Faculty of Mechanical Engineering, VSB-Technical University of Ostrava, 17. Listopadu 2172/15, 70800 Ostrava, Czech Republic; miroslav.mahdal@vsb.cz; 4 Centre for Control Systems, Vel Tech University, 400 Feet Outer Ring Road, Vel Nagar, Avadi, Chennai 600062, Tamil Nadu, India

**Keywords:** 2L−3PVSI inverter, 3L−3PNPC inverter, cost function, finite set model predictive control, incremental conductance, maximum power point tracking, photovoltaic systems

## Abstract

The Single-Stage Grid-Connected Solar Photovoltaic (SSGC-SPV) topology has recently gained significant attention, as it offers promising advantages in terms of reducing overall losses and installation costs. We provide a comprehensive overview of the system components, which include the photovoltaic generator, the inverter, the Incremental Conductance Maximum Power Point Tracking (IC-MPPT) algorithm, and the PI regulator for DC bus voltage control. Moreover, this study presents detailed system configurations and control schemes for two types of inverters: 2L−3PVSI and 3L−3PNPC. In order to perform a comparative study between the two structures, we subjected them to the same irradiation profile using the same grid configuration. The Photovoltaic Array (PVA) irradiance is increased instantaneously, in 0.2 s, from 400 W/m^2^ to 800 W/m^2^, is kept at 800 W/m^2^ for 0.2 s, is then gradually decreased from 800 W/m^2^ to 200 W/m^2^ in 0.2 s, is then kept at 200 W/m^2^ for 0.2 s, and is then finally increased to 1000 W/m^2^ for 0.2 s. We explain the operational principles of these inverters and describe the various switching states involved in generating output voltages. To achieve effective control, we adopt the Finite Set–Model Predictive Control (FS-MPC) algorithm, due to the benefits of excellent dynamic responsiveness and precise current tracking abilities. This algorithm aims to minimise the cost function, while taking into account the dynamic behaviour of both the PV system and the inverter, including any associated delays. To evaluate the performance of the FS-MPC controller, we compare its application in the three-level inverter configuration with the two-level inverter setup. The DC bus voltage is maintained at 615 V using the PI controller. The objective is to achieve a Total Harmonic Distortion (THD) below 5%, with reference to the IEEE standards. The 2L−3PVSI inverter is above the threshold at an irradiance of 200 W/m^2^. The 3L−3PNPC inverter offers a great THD percentage, meaning improved quality of the power returned to the grid.

## 1. Introduction

Competing and surviving in today’s challenging world requires balancing economic development with environmental conservation. Renewable energy sources (RESs) play a crucial role in achieving this balance. People nowadays are increasingly interested in utilising the vast potential of various RESs, such as solar and wind energy. The development of renewable energy generation has brought about a significant change in the energy sector. At present, renewable energy sources (RESs) meet approximately 17% of the global energy demand, and this figure is projected to rise in order to mitigate the adverse effects associated with conventional fossil fuel-based energy sources [1]. Traditional energy sources, such as petroleum and natural gas, are being depleted rapidly, leading to scarcity. As a result, there has been an increasing trend toward the use of non-traditional energy sources. Conventional energy sources have been depleted to a great extent due to their continued use, which has also contributed significantly to pollution and global warming. Therefore, scientists are emphasising the use of RESs. Non-traditional renewable energy sources are energy sources that are naturally replenished and do not run out [2].

In the past ten years, there has been a notable surge in the adoption of distributed energy resources, including solar photovoltaic systems (SPVSs) and energy storage systems (ESSs), in electrical power grids. Integrating these distributed energy resources into the power grid has brought several benefits, such as support during heavy loads and improved power quality. For example, utility-scale solar inverters have the ability to inject reactive power into the system to enhance the voltage profile, while ESSs can maintain the grid’s voltage and frequency during faults, allowing microgrids to continue providing power to loads [3,4]. Of all the solar technologies available, SPVSs are considered a promising option. Such systems can be managed either through a storage system or by being connected to the grid [5,6]. SPVSs are one of the most rapidly growing RESs. Multilevel inverters have gained interest for use in grid-interactive SPVSs because of their widespread use and strict grid codes [7]. For grid-tied SPVSs, various multilevel inverter topologies have been presented [7,8]. These inverters’ main goals are to feed the grid with as much active power as possible that has been extracted from the PVA. For the inverter to function properly, the specific goals of the various topologies of multilevel inverters (challenges arise in achieving DC-link voltage balancing in various topologies, such as neutral-point-clamped (NPC), cascaded H-bridge, and flying capacitor configurations) are also crucial [9]. Various traditional control methods, along with modulation techniques, have been discussed in the literature. However, controlling multiple objectives with classical controllers can be quite complicated [10].

Recently, Finite Set–Model Predictive Control (FS-MPC) has become widely popular in the field of power converter control, owing to its numerous benefits. It offers rapid dynamic response, stability, and precise control during steady-state operation. Moreover, FS-MPC allows for the integration of system nonlinearities and constraints into the control algorithm [11,12]. FS-MPC follows a unique approach that involves utilising a system model to predict the future behaviour of states within a specific time interval [11]. These predictions are then evaluated using a cost function, and the sequence that best minimises the cost function is selected to determine future control actions. Only the first value in the sequence is implemented, and the algorithm is recalculated for each sampling period.

Finite Set–Model Predictive Control (FS-MPC) possesses several advantages, such as its ability to effectively handle nonlinearities and constraints. However, it also has limitations due to the extensive computational requirements for solving the online optimisation problem, making it impractical when using the short sampling times typically employed in converter control. To mitigate this challenge, a potential solution is to solve the optimisation problem offline, as has been demonstrated in previous studies [13,14].

In this paper, we introduce a simplified configuration known as the Single-Stage Grid-Connected Solar Photovoltaic System (SSGC-SPVS). The system consists of a PVA, which can be configured in parallel or series depending on the desired voltage and power, connected to the grid through an inverter. The inverter can either be a three-level, three-phase neutral point clamped inverter (3L-3PNPC) or a two-level, three-phase voltage source inverter (2L−3PVSI). To optimise power extraction, we employ an incremental conductance algorithm (IC-MPPT) along with PI control to regulate the DC-bus voltage. A reference current is generated for the FS-MP controller, and the magnitude and frequency of the currents are determined using the FS-MPC algorithm.

The assessment of power quality being fed back into the grid is determined by analysing the THD of the grid currents. In previous research, when the irradiance fell under 500 W/m^2^, the THD of the grid currents got closer to the permissible threshold. For the 3L−3PNPC configuration, in reference [15], the THD was reported to be approximately 3.2% when exposed to an irradiance of 400 W/m^2^; meanwhile, in reference [16], under an irradiance of 800 W/m^2^, the THD reached 3.52%, and in reference [17], at an irradiance of 1000 W/m^2^, it dropped to 1.57%. For the 2L−3PVSI structure, in [18], at an irradiance of 1000 W/m^2^, the THD was reported to be 2.54%, while in [19], it was reported to be 1.4% at 1000 W/m^2^.

In this study, we propose the use of predictive control to supervise the inverter, with the aim of minimising the cost function while taking into consideration the dynamics of both the photovoltaic system, which experiences rapid changes in insolation, and the inverter, including any potential delays this may introduce. We provide a comprehensive overview of the overall system, including the two structures (2L−3PVSI and 3L−3PNPC), the PVA model, and the inverter topology. Furthermore, we detail the hierarchical control system, starting with IC-MPPT, DC bus, and MPC design. To evaluate the system’s performance, simulations are conducted using MATLAB and Simulink for both structures. The THD is assessed at different levels of irradiation—specifically, at 200 W/m^2^, 400 W/m^2^, 800 W/m^2^, and 1000 W/m^2^ for both structural configurations (2L−3PVSI and 3L−3PNPC). These data serve as the basis for a comparative analysis of the two structures. The results are thoroughly analysed and interpreted. Finally, the study concludes with a summary of the key research findings.

## 2. System Description

### 2.1. Global System Configuration

**Figure 1:**
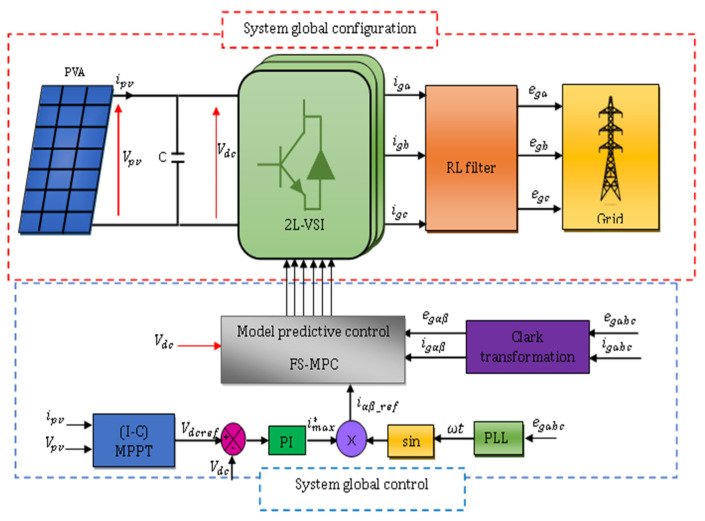
Global system configuration and control for the 2L−3PVSI structure.

**Figure 2:**
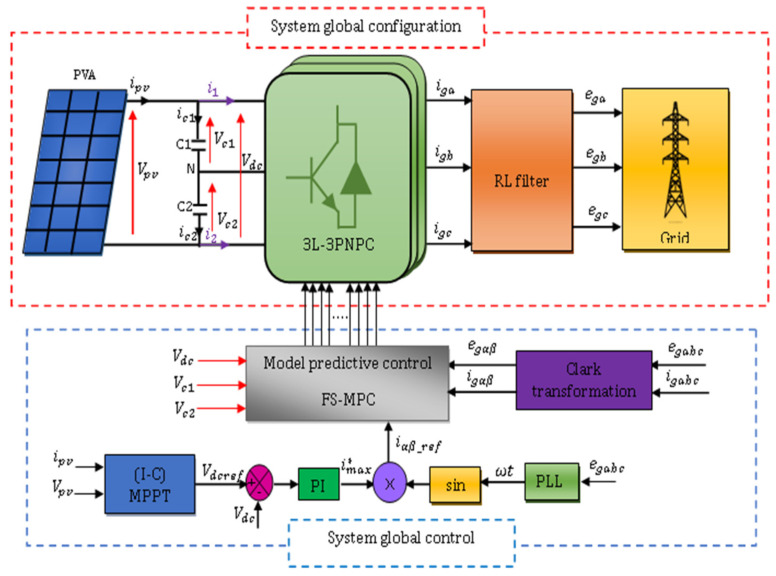
Global system configuration and control for the 3L−3PNPC structure.

**Figure 3:**
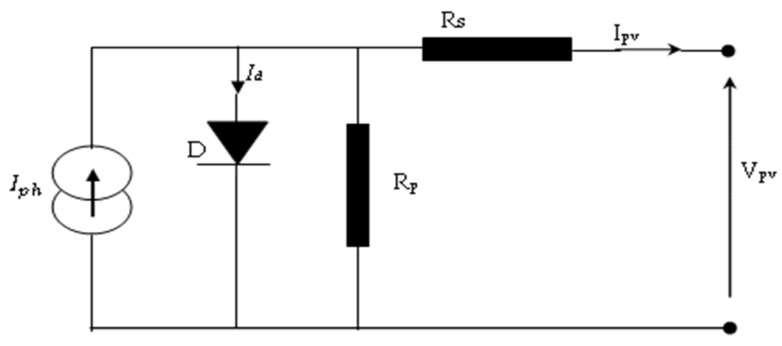
Single-diode model of PVA.

The general block diagram adopted for this study is a PVA connected through a single-stage grid-tied inverter in two configurations: 2L−3PVSI inverter (Figure 1) and 3L−3PNPC inverter (Figure 2) configuration. Figure 1 depicts the suggested model for a grid-tied 2L−3PVSI system. This model includes a PV panel group, a PLL circuit, an LR filter, and an IC-MPPT. Additionally, a block strategy controller is employed. The PV panel group is directly linked to the grid through the 2L−3PVSI inverter. The PLL circuit is employed to synchronise the 2L−3PVSI inverter output current with the grid voltage. Figure 2 depicts a 3L−3PNPC inverter configuration with two capacitors and a neutral clamped point. The IC-MPPT technique’s output establishes the reference voltage ($V_{dcref}$). The measured input voltage of the NPC ($V_{dc}$) is compared to this voltage, and one of the recommended control strategies is employed to generate the required reference current ($i_{max}^{*}$) in consideration of the resulting error.

### 2.2. PVA Configuration

The five-parameter single-diode model is widely recognised and valued for its simplicity and accuracy in modelling photovoltaic (PV) cells. One significant aspect of this model, as depicted in Figure 3, is the inclusion of parallel resistance (R_p_). This parameter is responsible for capturing the influence of factors such as leakage current, impurities, and crystal imperfections within the PV cell structure [20].

The output current of a solar cell, which includes the photocurrent, can be mathematically modelled by considering components such as light-generated current sources, diodes, and series and parallel resistances.

(1) $$I_{pv}=I_{ph}-I_{d}\left[\exp\left(\frac{q}{c_{B}TA}V_{pv}\right)-1\right]$$

(2) $$I_{ph}=G\left[I_{scr}+K_{i}\left(T-T_{r}\right)\right]$$

(3) $$I_{d}=I_{o}{\left[\frac{T}{T_{r}}\right]}^{3}\exp\left[\frac{qE_{g}}{KQA}\left(\frac{1}{T_{r}}-\frac{1}{T}\right)\right]$$

**Figure 4:**
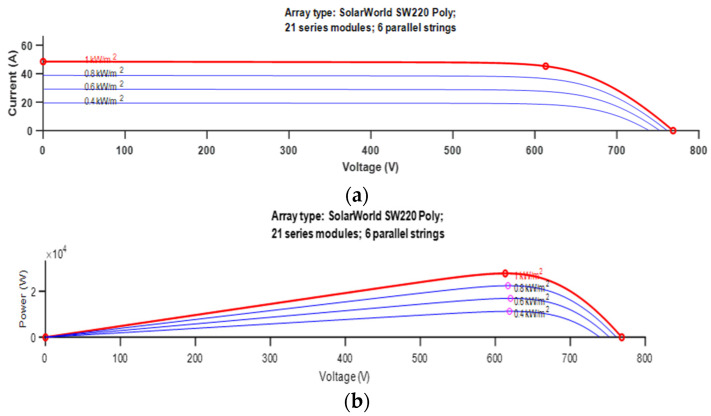
Characteristic of Solar World SW220 Poly and (**a**) Characteristic I/V and (**b**) Characteristic P/V.

Here, $I_{pv}$ is the output current and $V_{pv}=(A,V)$ is the output voltage, T is the temperature, G is the solar irradiance (W/m^2^), $I_{d}$ is PV saturation current, $I_{o}$ is the saturation current at $T_{r},$$I_{scr}$ is the short current under reference conditions, $T_{r}$ is the reference temperature, q is the electron charge, and $C_{B}$ is Boltzmann’s constant. The characteristics I/V and P/V of Solar World SW220 Poly are shown in Figure 4.

PV cells are placed into PV modules, which are organised into larger PV arrays. Achieving high efficiency from PV cells is crucial, but is often limited by financial constraints, resulting in an efficiency range of 9–20% [21]. PVA electricity generation depends on atmospheric conditions, with the I-V curve being nonlinear and influenced by solar irradiance changes, as shown in Figure 4a. Only the knee operation point in Figure 4b provides maximum power, and so it is essential to operate the PV generator at this point.

### 2.3. Inverter Configuration

The grid-tied inverters that we employed for our investigation have typical setups. The switching sequences and the functional schemes are defined for the two configurations.

#### 2.3.1. 2L-3PVSI Configuration

**Figure 5:**
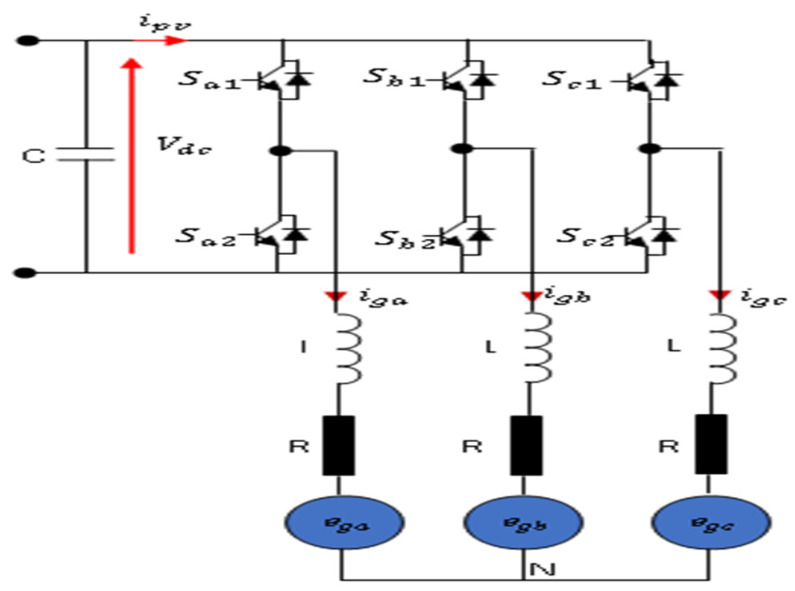
2L−3PVSI configuration.

The configuration of the 2L-3PVSI converter is illustrated in Figure 5. One crucial requirement for the converter’s optimal operation is to ensure that the switches in each leg operate in a complementary manner. This complementary mode of operation is essential for preventing any potential short circuits in the DC source. As a result, the converter is limited to a total of eight permissible switching states. Each of these switching states generates specific line-to-line output voltages and the DC-link current [22,23].

**Figure 6:**
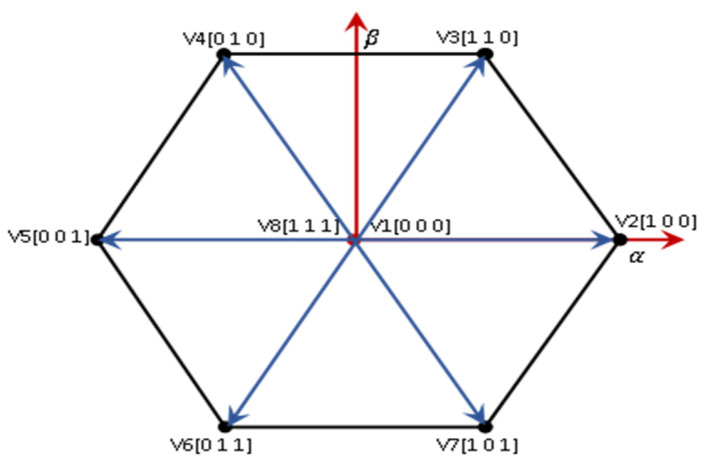
Voltage vectors in the complex plane of 2L−3PVSI inverter.

Figure 6 shows the eight switching states in the voltage vector topology on a complex plane.

The voltage vectors in Figure 6 can be described as follows.

(4) $$\left\{\begin{array}{l} V_{1}=0,\;V_{2}=\frac{2}{3}V_{dc},\;V_{3}=\left(\frac{1}{3}+j\frac{\sqrt{3}}{3}\right)V_{dc},\\ V_{4}=\left(-\frac{1}{3}+j\frac{\sqrt{3}}{3}\right)V_{dc},\;V_{5}=-\frac{2}{3}V_{dc},V_{6}=-\frac{1}{3}V_{dc}-j\frac{\sqrt{3}}{3}V_{dc},\\ V_{7}=\left(\frac{1}{3}\right)V_{dc},\;V_{8}=0 \end{array}\right.$$

#### 2.3.2. 3L-3PNPC Configuration

**Figure 7:**
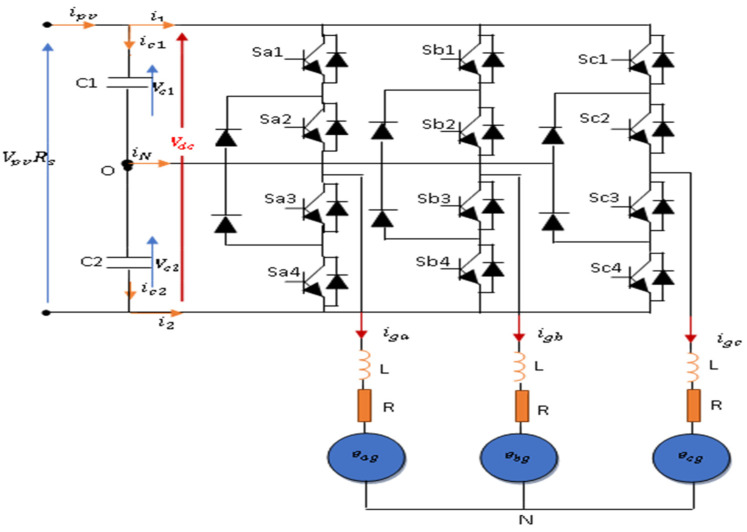
3L−3PNPC configuration.

NPC multilevel inverters are designed to generate a stepped output voltage waveform by utilising different levels of DC capacitor voltage [24]. For example, an m-level NPC inverter comprises (m^−1^) capacitors connected to the DC bus, 2 × (m^−1^) switching devices per phase, and 2 × (m^−2^) clamping diodes per phase. Figure 7 provides a visual representation of a three-level NPC inverter. To achieve this configuration, the DC bus voltage is divided into three distinct levels using two DC capacitors, namely, C1 and C2. Each capacitor maintains a voltage of V_DC_⁄2 volts, and the voltage distribution is limited to specific capacitor levels [24].

The utilisation of NPC multilevel inverters allows for the generation of output voltages with enhanced resolution and reduced harmonic distortion. By employing multiple capacitor levels, the staircase waveform can approximate a sinusoidal waveform with increased precision. This improved voltage quality is particularly advantageous in a variety of applications, including renewable energy systems and motor drives, as it helps minimise power losses and mitigate undesirable effects on connected devices.

The switching states of 3L-3PNPC are presented in Table 1.

In equation form, the following can be expressed:

(5) $$V_{xn}=\{\begin{array}{l} \begin{array}{ccc} V_{c1}+V_{c2} & \mathrm{if} & (S_{x1},S_{x2})\;\mathrm{are}\;\mathrm{ON} \end{array}\\ \begin{array}{ccc} V_{c2}\;\;\;\;\;\;\;\;\;\;\; & \mathrm{if} & (S_{x2},S_{x3})\;\mathrm{are}\;\mathrm{ON} \end{array}\\ \begin{array}{ccc} 0\;\;\;\;\;\;\;\;\;\;\;\;\;\;\; & \mathrm{if} & (S_{x3},S_{x4})\;\mathrm{are}\;\mathrm{ON} \end{array} \end{array}$$

(6) $$i_{xn}=\{\begin{array}{lll} i_{1} & \;\mathrm{if}\; & (S_{x1},S_{x2})\;\mathrm{are}\;ON\\ i_{N} & \;\mathrm{if}\; & (S_{x2},S_{x3})\;\mathrm{are}\;ON\\ i_{2} & \;\mathrm{if}\; & (S_{x3},S_{x4})\;\mathrm{are}\;\mathrm{ON}\; \end{array}$$

**Figure 8:**
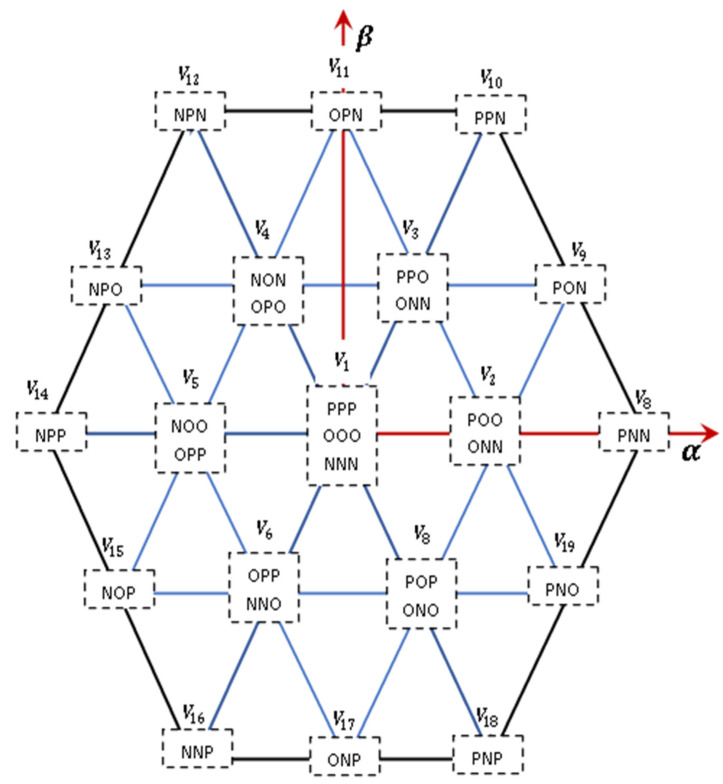
Voltage vectors and switching states in a 3L−3PNPC inverter.

Figure 8 illustrates the potential voltage vectors and corresponding switching states.

## 3. System Control

The control system is divided into three stages for the two configurations (i.e., 2L-3PVSI and 3L−3PNPC). The first is the IC-MPPT, the second is the DC-voltage control, followed by, finally, FS-MPC.

### 3.1. IC-MPPT Algorithm

**Figure 9:**
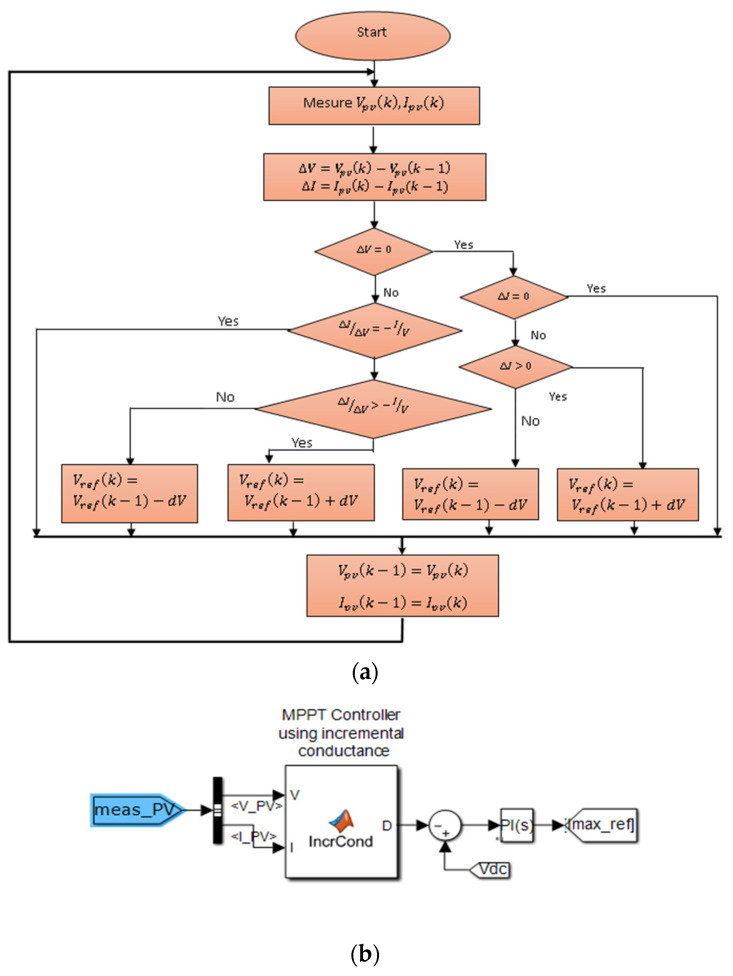
(**a**) Flowchart of the IC-MPTT algorithm; (**b**) block diagram for IC-MPPT using MATLAB/Simulink.

In order to optimise the energy output of a photovoltaic (PV) system in variable weather conditions, it is essential to incorporate a maximum power point tracking (MPPT) algorithm. The IC-MPPT algorithm is based on the concept of utilising the incremental conductance of the PV panel to determine the slope of the power curve. By ensuring that the incremental conductance matches its instantaneous value, the MPPT algorithm effectively tracks the maximum power point [24,25]. Figure 9 provides a visual representation of the flowchart for the IC-MPPT algorithm.

### 3.2. DC-Bus Voltage Control

The DC-bus voltage is maintained at its reference level for the two configurations, i.e., for both 2L−3PVSI and 3L−3PNPC inverters. For 3L−3PNPC inverter, the measured DC-bus voltage is the sum of the two capacitors.

**Figure 10:**
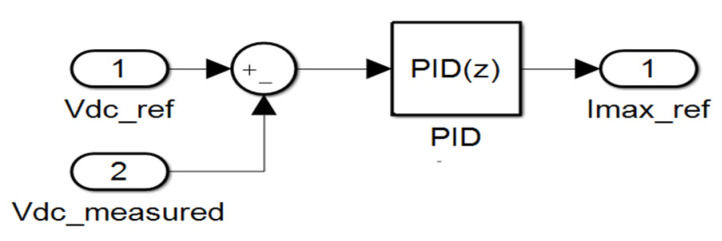
PI corrector for the DC-bus voltage.

A simple PI regulator is used in the two configurations, as shown in Figure 10. The output of the PI controller is the amplitude reference current, which constitutes the input of the model’s predictive controller.

### 3.3. MP Controller Design

Finite Set–Model Predictive Control (FS-MPC) is a highly popular approach employed in power electronic converters to effectively manage the flow of electrical energy. This technique is renowned for its advantages, which include its simple design and remarkable dynamic performance [26,27]. The underlying principle of FS-MPC revolves around selecting the most suitable switching state of the power converter in order to minimise the future deviation of the controlled variable [28,29].

During the implementation of Finite Set–Predictive Model Control (FS-MPC), an important aspect to consider is the evaluation of the cost function. This function considers different terms that are derived from the controlled variables and operating conditions. To achieve the desired performance, it is crucial to define weighting factors that establish the relationship between these terms. Nonetheless, a significant challenge in deploying FS-MPC is the careful selection of appropriate weighting factors that can adequately balance the control objectives. This task requires finding the optimal combination of weights to assign to various terms in the cost function, which is essential for achieving the desired control performance. This issue has been addressed in previous studies [29,30].

The FS-MPC algorithm for the control of the 3L-NPC is initialised with the discretisation of DC current. The equations governing the dynamic behaviour of the voltage across the DC-link capacitor can be expressed as follows:

(7) $$\frac{dV_{c1}}{dt}=\frac{1}{C}i_{c1}(t)$$

(8) $$\frac{dV_{c2}}{dt}=\frac{1}{C}i_{c2}(t)$$

Here, $C_{1}$ and $C_{2}$ are capacitances across the upper and lower DC-link capacitors, respectively. Additionally, $V_{c1}$ and $V_{c2}$ are DC-link capacitor voltages. Moreover, $i_{c1}$ and $i_{c2}$ are currents through capacitors $C_{1}$ and $C_{2},$ respectively.

To predict and anticipate the dynamics of the variables involved in the cost function, it is essential to utilise a discrete-time model of the system. This discrete-time model allows for the formulation of mathematical equations that describe the system’s behaviour over discrete time intervals. To achieve this, the Euler preview technique is employed due to its simplicity and acceptable accuracy, which is very important for achieving improved performance [23,31]. Using this technique, the system’s discrete time form can be obtained, as shown in the following:

(9) $$\frac{di(t)}{dt}\approx \frac{i(k+1)-i(k)}{T_{s}},$$

where $T_{s}$ is the sampling period.

Euler’s method is utilised to discretise Equation (9), resulting in an equation that enables the prediction to anticipate the forthcoming current at (k+1)−th time step for the 27 potential switching states applied to the inverter. The resulting relation is presented in Equation (10) in the following format:

(10) $$\frac{dV_{cx}}{dt}\approx \frac{V_{cx}(k+1)-V_{cx}(k)}{T_{s}}$$

The discrete-time equations that provide the predicted values of $V_{c1}^{p}\left(k+1\right)$ and $V_{c2}^{p}\left(k+1\right)$ are stated as follows:

(11) $$V_{c1}^{p}(k+1)=V_{c1}(k)+\frac{T_{s}}{C}i_{c1}(k)$$

(12) $$V_{c2}^{p}(k+1)=V_{c2}(k)+\frac{T_{s}}{C}i_{c2}(k)$$

Equations (13) and (14) indicate that the input current and the steady states of the inverter have an impact on the currents $i_{c1}$ and $i_{c2}.$

(13) $$i_{c1}(k)=i_{dc}(k)-\left[K_{1a}i_{a}(k)+K_{1b}i_{b}(k)+K_{1c}i_{c}(k)\right]$$

(14) $$i_{c2}(k)=i_{dc}(k)-\left[K_{2a}i_{a}(k)+K_{2b}i_{b}(k)+K_{2c}i_{c}(k)\right]$$

The current values $i_{c1}(k)$ and $i_{c2}(k)$ depend on the value of the input currents and switching states of the inverter, as expressed in Equations (13) and (14). We note that $i_{dc}(k)$ is the current furnished by the voltage source $V_{dc}.$ Furthermore, the values of $K_{1x}$ and $K_{2x}$ depend on the switching states.

(15) $$K_{1x}=\{\begin{array}{l} \begin{array}{cc} 1 & \mathrm{if}\;S_{x}=(+) \end{array}\\ \begin{array}{cc} 0 & \mathrm{otherwise} \end{array} \end{array}\;(\mathrm{where}\;x=a,b,c)$$

(16) $$K_{2x}=\{\begin{array}{l} \begin{array}{cc} 1 & \mathrm{if}\;S_{x}=(-) \end{array}\\ \begin{array}{cc} 0 & \mathrm{otherwise} \end{array} \end{array}\;(\mathrm{where}\;x=a,b,c)$$

The characteristics of the grid current flowing through the interfacing filter inductor can be described as follows:

(17) $$L\frac{di_{gx}(t)}{dt}=V_{invx}(t)-Ri_{gx}(t)-e_{gx}(t),$$

where $V_{invx}$ is the inverter voltage output.

The three-phase abc- reference frame variables $(e_{gx}$ and $i_{gx})$ are transformed into an orthogonal αβ- reference frame ${(e}_{g\alpha \beta }$ and $i_{g\alpha \beta })$ using Clarke’s transformation matrix (P), given as follows:

(18) $$P=\left[\begin{array}{ccc} 1 & -\frac{1}{2} & -\frac{1}{2}\\ 0 & \frac{\sqrt{3}}{2} & -\frac{\sqrt{3}}{2} \end{array}\right]$$

The discrete-time model of the grid current is obtained as follows:

(19) $$i_{g\alpha \beta }(k+1)=\left(1-\frac{RT_{s}}{L}\right)i_{g\alpha \beta }(k)+\frac{T_{s}}{L}\left[V_{inv\alpha \beta }(k)-e_{g\alpha \beta }(k)\right]$$

**Figure 11:**
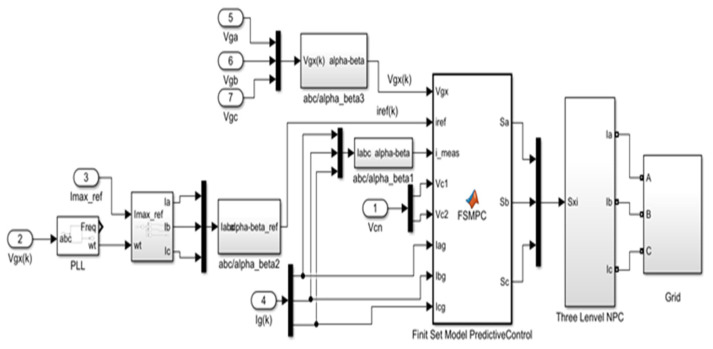
Block diagram for FS-MPC-based 3L−3PNPC inverter implementation using MATLAB/Simulink.

Here, $V_{inv\alpha \beta }\left(k\right)$ is the evaluated voltage vector, which belongs to all 19 voltages. Figure 11 illustrates the predictive control strategy employed for the 3L−3PNPC inverter implemented in MATLAB/Simulink. In this approach, future values of current and potential differences across the capacitors are predicted using measurements taken from the inverter.

A total of 27 switching states are generated, and corresponding cost functions are evaluated based on the predictions obtained. Transition states that reduce the cost function J are selected and applied in the next sampling stage.

In order to minimise the difference between the measured current and the desired value, the Neutral Point Clamped (NPC) inverter employs a cost function (J) as a reference value. This cost function is specifically designed to quantify the extent of the deviation between the actual and desired currents.

By utilising the cost function given by Relation (20), the NPC inverter aims to optimise its control strategy and adjust its operation to achieve a current output that closely matches the desired waveform. The formulation of the cost function J  takes various factors into consideration, such as system constraints, performance objectives, and control requirements.

The cost function *J* is defined as follows:

(20) $$J=\left|i_{\alpha }^{ref}\left(k+1\right)-i_{\alpha }^{p}\left(k+1\right)\right|+\left|i_{\beta }^{ref}\left(k+1\right)-i_{\beta }^{p}\left(k+1\right)\right|+\gamma \left|V_{c1}^{p}\left(k+1\right)-V_{c2}^{p}(k+1)\right|$$

Here, $i_{\alpha }^{ref}\left(k+1\right),$ $i_{\alpha }^{p}\left(k+1\right)\;$ and $i_{\beta }^{ref}\left(k+1\right),\;i_{\beta }^{p}(k+1)$ indicate the real and imaginary parts of the reference and predicted currents in the αβ  frame. The symbols $V_{c1}^{p}\left(k+1\right),\;V_{c2}^{p}(k+1)$ represent the anticipated values of the DC-link capacitor voltages. Additionally, γ is the weighting factor (γ=0.001).

**Figure 12:**
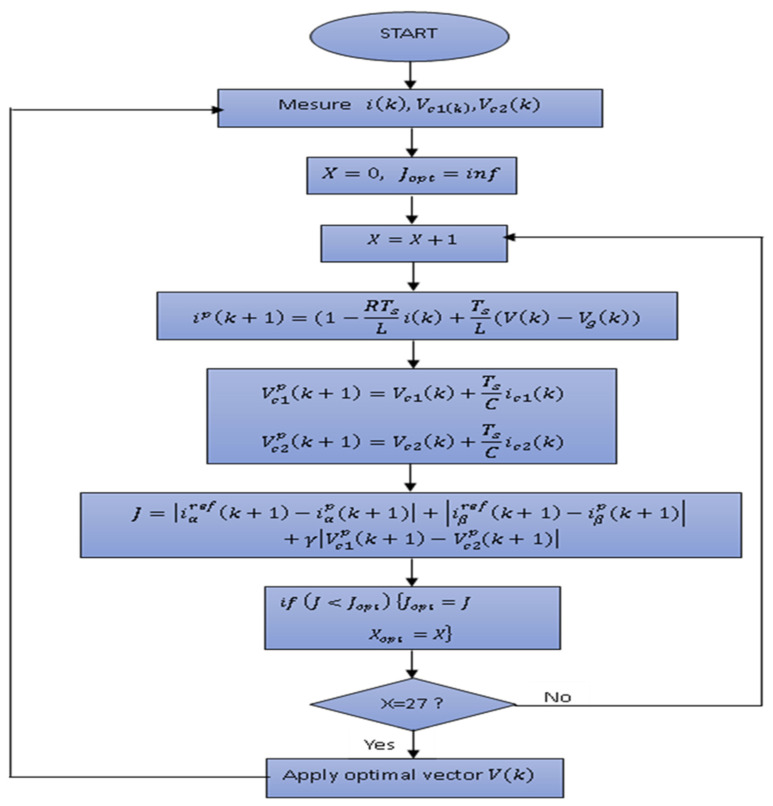
Flowchart of the MPC for the 3L−3PNPC inverter.

Figure 12 presents the flowchart illustrating the various steps of the MPC algorithm for the 3L-3PNPC inverter.

**Figure 13:**
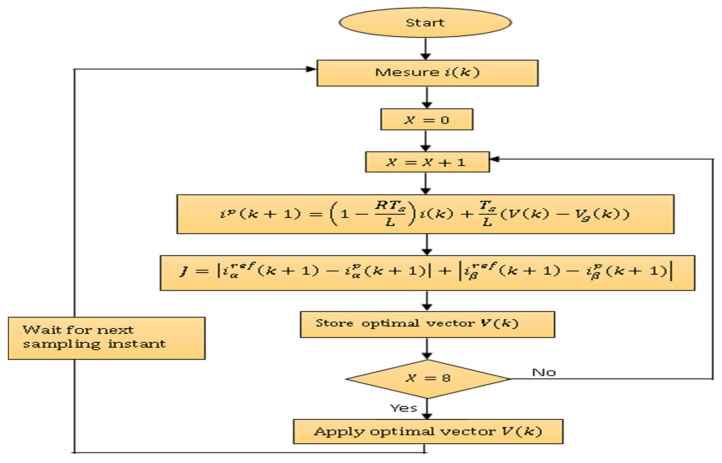
Flowchart of the MPC for the 2L−3PVSI Inverter.

For the 2L-3PPVSI, the same algorithm is applied, in just one difference in the capacitor voltage, which do not constitute an input of the MP Controller. Figure 13 presents a flowchart for the MPC algorithm for the 2L−3PVSI inverter.

## 4. Simulation Results

To conduct a comparative analysis between a photovoltaic generator interfaced with the electrical grid using a two-level inverter (2L-3PVSI) and a three-level inverter (3L−3PNPC), we employed MATLAB Simulink to model both structures. The objective was to evaluate their performance under identical conditions, utilising the same PVA and electrical grid configuration.

For the control of both structures, we opted for the Finite Set Model Predictive Control (FS-MPC) technique. This control strategy was chosen due to its desirable features, which include simplicity and excellent dynamic responsiveness.

To ensure a fair comparison, we employed the same simulation parameters for both structures. These parameters, outlined in Table 2, encompass various aspects related to the simulation setup, such as time steps, sampling rates, and system configurations.

**Figure 14:**
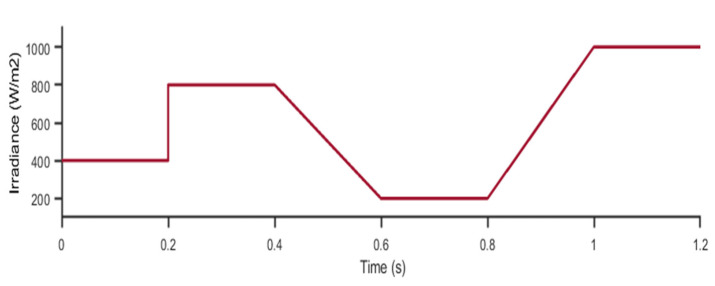
Irradiance for the 2L−3PVSI and 3L−3PNPC (W/m^2^).

Figure 14 displays the irradiance profile of photovoltaic solar panels (PV) during a typical day. This irradiance profile is crucial for determining the availability of the solar energy captured by the PV panels.

**Figure 15:**
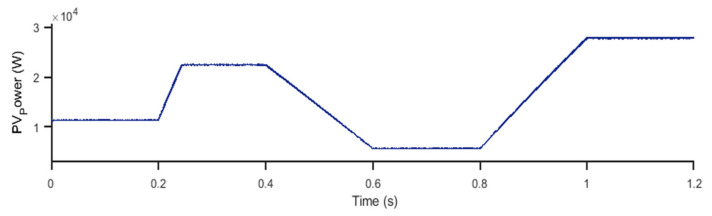
PV power for 2L−3PVSI and 3L−3PNPC structures.

Figure 15 illustrates the power delivered by the photovoltaic solar panels (PV) determined on the basis of the irradiance profile presented in Figure 14.

**Figure 16:**
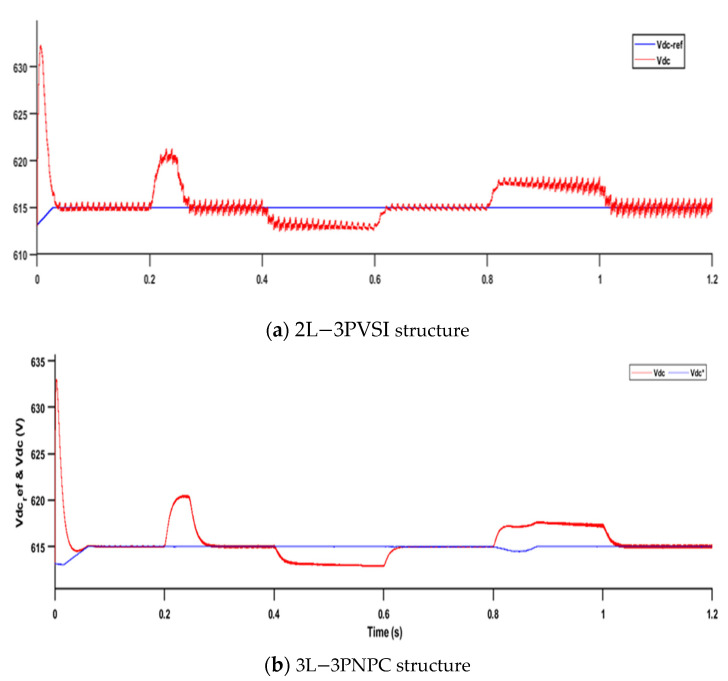
DC bus measured and reference voltages.

Figure 16 presents the results of the measured and reference voltages of the DC bus for two different structures: (a) 2L−3PVSI and (b) 3L−3PNPC.

**Figure 17:**
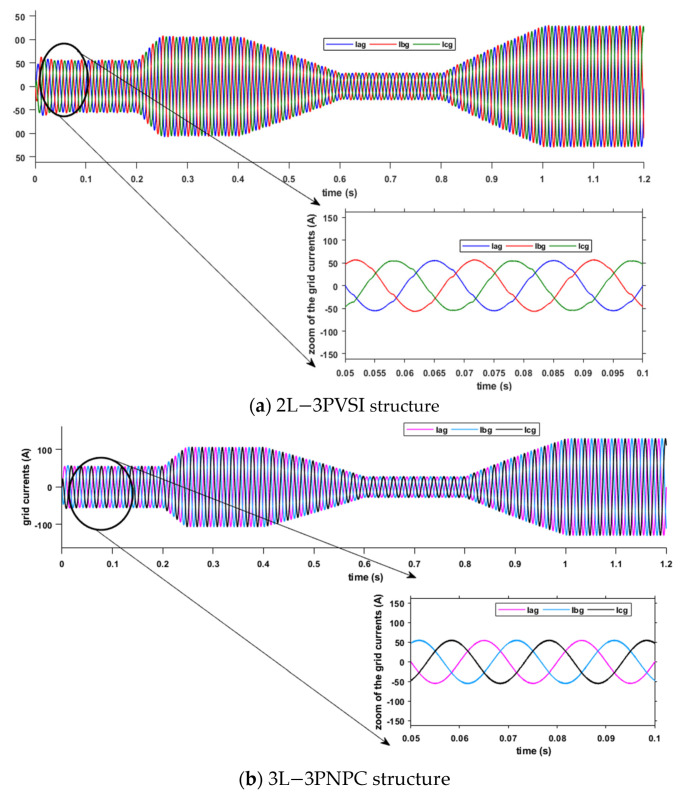
Inverter grid current.

Figure 17 displays the results of the grid current for two different structures: (a) 2L−3PVSI and (b) 3L−3PNPC.

**Figure 18:**
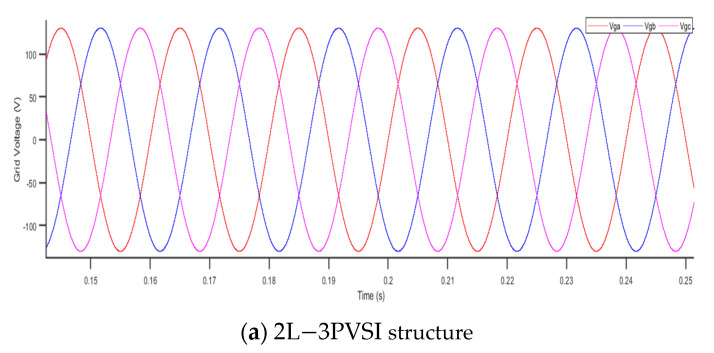

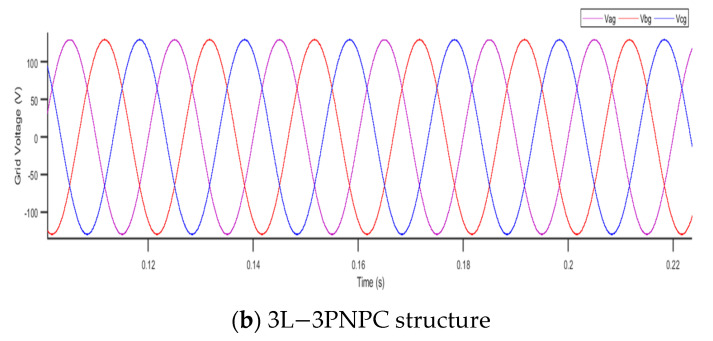
Grid voltage.

Figure 18 presents the results of the grid voltage for two different structures: (a) 2L−3PVSI and (b) 3L−3PNPC.

**Figure 19:**
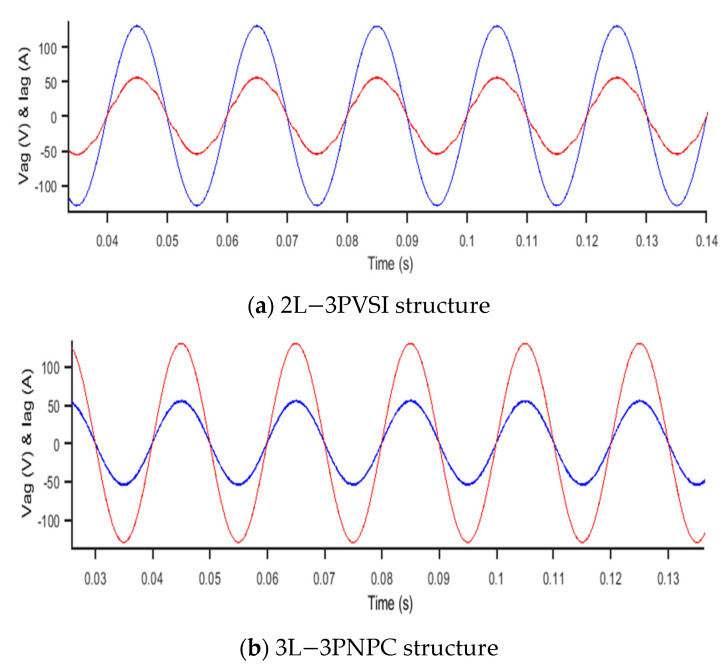
Phase voltage and grid current.

Figure 19 presents the results of network voltage and phase current for two different structures: (a) 2L−3PVSI and (b) 3L-3PNPC.

**Figure 20:**
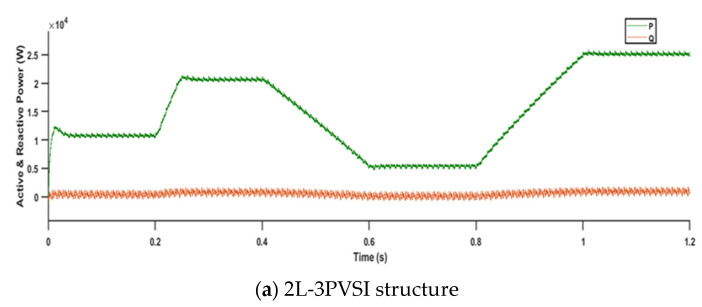

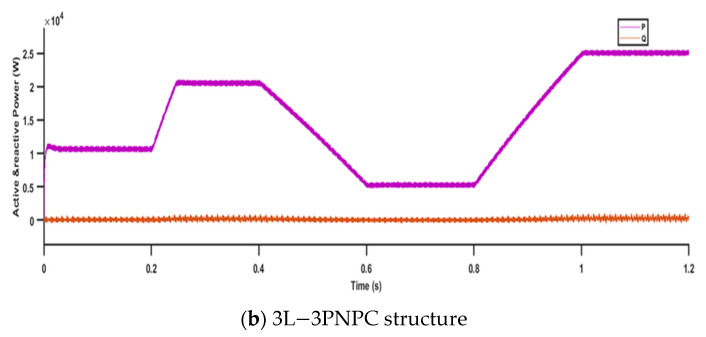
Active and reactive power.

Figure 20 presents the results of active and reactive power for two different structures: (a) 2L−3PVSI and (b) 3L−3PNPC.

**Figure 21:**
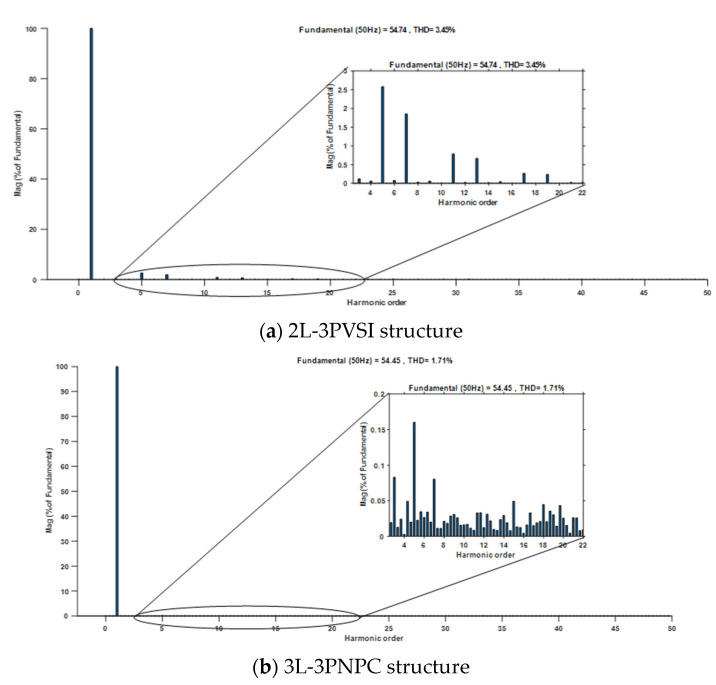
Grid current THD and its zoom at irradiance = 400 W/m^2^.

Figure 21 displays the total harmonic distortion (THD) of the grid current for two different structures: (a) 2L−3PVSI and (b) 3L−3PNPC.

Table 3 shows the comparative analysis of the two structures in terms of power ripples, dynamic response, and total harmonic distortion.

## 5. Discussion

As shown in Figure 15, a direct correlation is observed between the solar irradiance profile and the power delivered by the PV panels. When solar irradiance is high, the generated power reaches its maximum, while during periods of low irradiance, the generated power decreases. This close correlation between the solar irradiance profile and the generated power confirms that the photovoltaic system perfectly follows the chosen irradiance profile, demonstrating the efficiency of converting solar energy into electricity by the PV panels.

In Figure 16, when the 2L-3PVSI structure is used, it can be observed that the fluctuations of the DC bus voltage are not perfectly reduced compared to the 3L-3PNPC structure. This indicates that the 3L−3PNPC structure is better at regulating the DC bus voltage, reducing undesirable voltage variations to a greater extent. On the other hand, the 2L-3PVSI structure shows a tendency to exhibit larger ripples in the DC bus voltage, which can have an impact on the stability and quality of the power supply system.

In Figure 17, when the 3L−3PNPC structure is employed, it can be noticed that the currents are perfectly sinusoidal compared to the 2L−3PVSI structure. This indicates that the 3L-3PNPC structure generates more regular and currents with higher quality, resulting in the injection of energy of excellent quality into the electrical grid. On the other hand, the 2L-3PVSI structure exhibits currents that may have distortions and harmonics, which can adversely affect the quality of the energy injected into the grid. Consequently, the utilisation of the 3L−3PNPC structure significantly enhances the quality of the energy supplied to the network.

When using the 2L−3PVSI structure, the 3L−3PNPC structure generates a more stable grid voltage that is closer to the reference value, as illustrated in Figure 18. The stability of the grid voltage is essential for ensuring reliable and optimal operation of electrical devices linked to the grid. Therefore, the use of the 3L−3PNPC structure can contribute to enhancing the quality of the electrical energy supplied to the network.

In Figure 19, both structures operate with a unity power factor, meaning that they inject active power equal to the apparent power into the grid. However, the 3L-3PNPC structure exhibits better stability in terms of grid voltage and phase current than the 2L-3PVSI structure. Stable grid voltage and phase current are crucial for maintaining the balance of the electrical grid and ensuring the proper operation of connected devices. Thus, the use of the 3L−3PNPC structure can provide improved stability and reliability when supplying electrical energy to the grid.

In Figure 20, For both structures, the active and reactive powers follow their respective references, indicating a balance between energy production and consumption. However, the 3L−3PNPC structure exhibits superior stability in terms of both active and reactive power, with no fluctuations, when compared to the 2L−3PVSI structure. A stable and ripple-free behaviour for active and reactive power is crucial for ensuring efficient and reliable system operation, as well as better energy management. Therefore, the use of the 3L-3PNPC structure ensures increased stability in active and reactive powers, ultimately enhancing the quality of the energy supplied to the grid.

**Figure 22:**
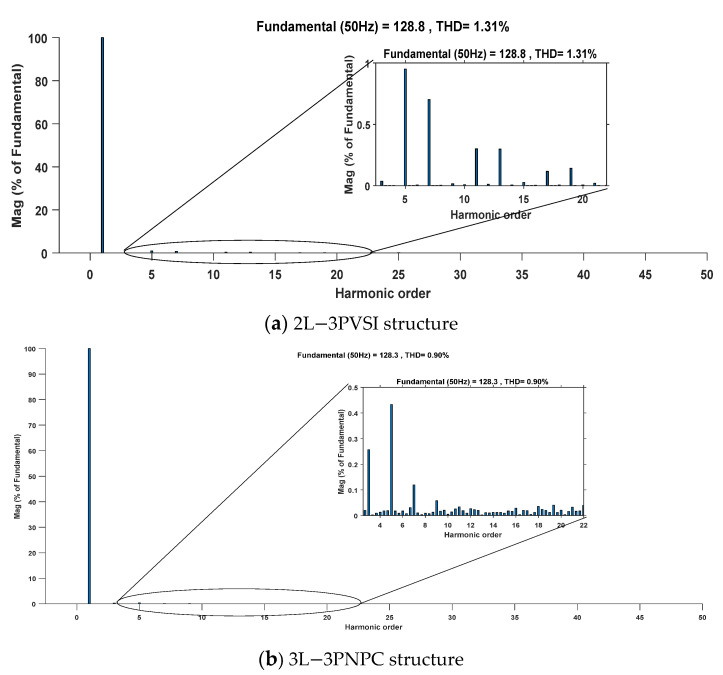
Grid current THD and its zoom at irradiance = 1000W/m^2^.

With a solar irradiance of 400 W/m^2^ it can be observed that the THD of the grid current is lower when the 3L−3PNPC structure is used, with a value of 1.75%, compared to the 2L-3PVSI structure, which exhibits a THD of 3.45%, as shown in Figure 21. This indicates that the 3L−3PNPC structure generates grid current with less harmonic distortion, resulting in improved quality of the injected electrical energy into the grid. However, with a solar irradiance of 1000 W/m^2^, the 3L−3PNPC structure once again demonstrates a significant improvement in terms of harmonic distortion of the current, as shown in Figure 22. Its THD is 0.90%, while the 2L−3PVSI structure has a THD of 1.31%. This difference highlights the superior ability of the 3L−3PNPC structure to generate cleaner grid current that closely approximates an ideal sinusoidal waveform, as detailed in Table 3. Table 3 also assesses both dynamic response and power fluctuations. The 3L−3PNPC configuration demonstrates superior dynamic performance when compared to the 2L-3PVSI setup. Specifically, for an irradiance range of 0 to 400 W/m^2^, the response time is reduced from 0.04 s (in the 2L−3PVSI structure) to 0.025 s (in the 3L−3PNPC structure). Similarly, for irradiance levels between 400 and 800 W/m^2^, the 3L−3PNPC setup achieves a response time of 0.015 s, whereas the 2L-3PVSI structure lags behind, with a response time of 0.07s. The 2L−3PVSI exhibits higher power fluctuations, whereas they are reduced when the irradiance is at 1000 W/m^2^. Specifically, at an irradiance of 1000 W/m^2^, the 3L−3PNPC exhibits a power fluctuation of 0.069 kW, whereas the 2L−3PVSI shows a slightly higher power fluctuation, at approximately 0.086 kW.

Table 4 illustrates that the THD results exhibit significant variations compared to those reported in references [18,19], with higher THD values in most cases for the two-level structure presented in this study. This may indicate a more substantial harmonic distortion in the grid current for the 2L−3PVSI configuration used. However, it is worth noting that these values remain within acceptable limits according to IEEE 512 standards.

Table 5 reveals that the THD results exhibit significant variation compared to those reported in references [15,16,17], with higher THD values in most cases for the three-level structure (3L−3PNPC) presented in this study. This may indicate a more substantial harmonic distortion in the grid current for the 3L−3PNPC configuration used. Nevertheless, these values remain within acceptable limits according to the IEEE 512 standards. The discrepancies between this study and the references for both structures (3L−3PNPC and 2L−3PVSI) can be attributed to differences in simulation parameters and component models.

## 6. Conclusions

This study aimed to assess the performance of two configurations for integrating solar energy into the electrical grid, namely, the two-level inverter system (2L−3PVSI) and the three-level NPC inverter system (3L−3PNPC), using a Finite Set Model Predictive Control (FS-MPC) approach. The results obtained clearly demonstrate that the 3L-3PNPC inverter has significant advantages in terms of the quality of the energy injected into the grid and dynamic performance compared to the 2L−3PVSI inverter. However, the 2L−3PVSI inverter still maintains its appeal in terms of cost and ease of modelling and control, especially for solar irradiance levels exceeding 400 W/m^2^.

Nevertheless, it is crucial to note that this study has limitations. The simplified models used for both the 2L−3PVSI and 3L−3PNPC inverters may not fully represent performance in real-world environments, and the lack of integration of grid disturbances is also a point to consider. Therefore, further research is needed to explore these systems more comprehensively, incorporating more detailed models and grid disturbance scenarios.

Future research required in this field includes the optimisation of control strategies for both configurations, taking into account dynamic variations in solar irradiance and grid disturbances. Additionally, experimental studies are necessary to validate simulation results and assess the actual performance of these systems. Finally, analysing the cost effectiveness and environmental impact of these configurations in real-world conditions is a promising research area for guiding the practical implementation of the integration of solar energy into electrical grids.

## Figures and Tables

**Table 1:** Switching states of an 3L−3PNPC inverter (x=a,b,c).

*S_x_*	*S*_*x*1_	*S*_*x*2_	*S*_*x*3_	*S*_*x*4_	*S*_*x*0_
+	1	1	0	0	VDC/2
0	0	1	1	0	0
−	0	0	1	1	−VDC/2

**Table 2:** PVA and grid parameters.

Parameters	Values
P_mpp_ (W)	220.168
V_mpp_ (V)	29.2
I_mpp_ (A)	7.54
Voc (V)	36.6
Isc (A)	8.08
Parallel string	6
Series modules	21
$R_{g}\;(\Omega )$	0.1
L_g_ (H)	3.3 × 10^−3^
Grid voltage (V)	130
f grid (HZ)	50
MPPT-Ts (s)	1 × 10^−5^
FS-MPC-Ts (s)	1 × 10^−5^
DC-Bus Capacitor (F)	2.2 × 10^−9^

**Table 3:** Comparative analysis of the two structures.

Irradiance (W/m^2^) = 200			
Structure	Power ripples (kW)	Dynamic response (s)	THD_i_ (%)
2L−3PVSI	0.09	N/A	6.83
3L−3PNPC	0.08	N/A	3.32
Irradiance (W/m^2^) = 400			
Structure	Power ripples (kW)	Dynamic response (s)	THD_i_ (%)
2L−3PVSI	0.088	0.04	3.45
3L−3PNPC	0.075	0.025	1.75
Irradiance (W/m^2^) = 800			
Structure	Power ripples (kW)	Dynamic response (s)	THD_i_ (%)
2L−3PVSI	0.087	0.07	1.66
3L−3PNPC	0.073	0.015	0.98
Irradiance (W/m^2^) = 1000			
Structure	Power ripples (kW)	Dynamic response (s)	THD_i_ (%)
2L−3PVSI	0.086	N/A	1.31
3L−3PNPC	0.069	N/A	0.9

**Table 4:** Grid current THD of the 2L-3PVSI structure in previous works.

2L-3PVSI				
Reference [18]				
Irradiance (W/m^2^)	1000	800	400	200
THD (%)	2.25	N/A	N/A	N/A
Reference [19]				
Irradiance (W/m^2^)	1000	800	400	200
THD (%)	1.40	1.60	2.6	6.1

**Table 5:** Grid current THD of 3L−3PNPC structure in previous works.

3L−3PNPC				
Reference [15]				
Irradiance (W/m^2^)	1000	800	400	200
THD (%)	0.97	1.51	3.2	N/A
Reference [16]				
Irradiance (W/m^2^)	1000	800	400	200
THD (%)	N/A	N/A	3.45	N/A
Reference [17]				
Irradiance (W/m^2^)	1000	800	400	200
THD (%)	1.57	N/A	N/A	N/A

## Data Availability

The data used in this paper can be obtained from the authors upon request.

## Abbreviations

2L-3PVSI	Two-Level, Three-Phase Voltage Source Inverter
3L-3PNPC	Three-Level, Three-Phase Neutral Point Clamped
DC	Direct Current
ESS	Energy Storage System
FS-MPC	Finite Set Model Predictive Control
IC	Incremental Conductance
MPPT	Maximum Power Point Tracking
PI	Proportional Integrator
PLL	Phase Looked Loop
PV	Photovoltaic
PVA	Photovoltaic Array
RES	Renewable Energy Source
SPVS	Solar Photovoltaic System
SSGC-SPVS	Single Stage Grid Connected Solar Photovoltaic System
THD	Total Harmonic Distortion
N/A	Note Applicated
